# What drives our aesthetic attraction to birds?

**DOI:** 10.1038/s44185-023-00026-2

**Published:** 2023-09-27

**Authors:** Andrea Santangeli, Anna Haukka, William Morris, Sarella Arkkila, Kaspar Delhey, Bart Kempenaers, Mihai Valcu, James Dale, Aleksi Lehikoinen, Stefano Mammola

**Affiliations:** 1 Animal Demography and Ecology Unit, Institute for Mediterranean Studies (IMEDEA), CSIC-UIB, 07190 Esporles, Spain; 2 Research Centre for Ecological Change, Organismal and Evolutionary Biology Research Programme, University of Helsinki, 00014 Helsinki, Finland; 3 The Helsinki Lab of Ornithology, Zoology Unit, The Finnish Museum of Natural History (LUOMUS), University of Helsinki, Helsinki, Finland; 4 Biodiversity Informatics Unit, Finnish Museum of Natural History (LUOMUS), University of Helsinki, Helsinki, Finland; 5 Max Planck Institute for Biological Intelligence, Seewiesen, Germany; 6 School of Natural and Computational Sciences, Massey University, Auckland, New Zealand; 7 Molecular Ecology Group (MEG), Water Research Institute (IRSA), National Research Council (CNR), Verbania Pallanza, Italy; 8 Laboratory for Integrative Biodiversity Research (LIBRe), Finnish Museum of Natural History (LUOMUS), University of Helsinki, Helsinki, Finland; 9 National Biodiversity Future Center, Palermo, Italy

**Keywords:** Biodiversity, Biogeography, Conservation biology, Macroecology, Anthropology, Cultural evolution, Society

## Abstract

In the Anthropocene, the era when the imprint of humans on nature is pervasive across the planet, it is of utmost importance to understand human relationships with other species. The aesthetics of nature, and of species, is one of the values that plays a role in shaping human-nature relationships. Birds are ubiquitous across the world. The beauty of birds exerts a powerful tug on human emotions, and bird-rich areas attract scores of eco-tourists. People naturally find some birds more beautiful or interesting than others, but we currently lack a global understanding of the specifics of what makes a species aesthetically attractive. Here, we used a global citizen-science database on bird attractiveness covering nearly all extant bird species, to show that there are specific visual features that drive our aesthetic appeal for some bird species over others. First, our aesthetic attraction is highest for smaller birds with specific, vivid colors (e.g., blue and red, and departing from brown-grey) and extreme ornaments (a long crest or tail). Second, our aesthetic attraction is highest for species with broad ranges, possibly because such species may be more familiar to us. The features that make us attracted to a particular bird strongly align with broad human visual aesthetic preferences in modern society. Unveiling the visual features underpinning our aesthetic attraction to birds is a critical step towards optimizing conservation (e.g., via conservation marketing) and education campaigns, and leverage the cultural ecosystem service potential of birds.

## Introduction

Humans have an intimate relationship with birds. Birds are very common in art (e.g., the Rime of the Ancient Mariner), they populate myths and religions (e.g., the bird-headed gods of Egypt), are an essential component of traditional/cultural knowledge^1^, and provide food and other services for billions of people^2,3^. In recent decades, our fascination for birds manifests in the widespread hobby of birdwatching, a multi-million-dollar global business^2^. When we are exposed to birds, beside cultural, emotional and past history effects that we might associate to a specific bird species, we also receive direct aesthetic stimuli (e.g. color, shape, size), in addition to bird sound, behavioral stimuli and numerous other traits that appeal to humans. The combination of the above stimuli contributes to shape our aesthetic value for different bird species. Given the universal interest in birds, it thus comes naturally to ask: What is it that makes some birds more aesthetically attractive than others to humans?

Determining human aesthetic preferences for birds is not only interesting to increase our understanding of human-nature relationships^4–6^, but is also relevant for conservation. Recent research shows that more aesthetically appealing (e.g., colorful) species may be under higher threat (e.g., due to wildlife trade^7^), but they also receive more research and conservation attention compared to less aesthetically attractive species^8–10^. While specific studies have attempted to quantify what features make a bird attractive to humans^8,11,12^, these have all been taxonomically and/or geographically restricted. Therefore, we are still lacking a global understanding of what features drive human’s visual attraction to birds.

Here we use a citizen science based dataset on human-derived visual aesthetic attraction scores for nearly all the bird species of the world^13^ to determine how certain colors and morphology related traits affect human aesthetic preferences for bird species. The largest advance that this study provides to the current knowledge is the scale of the investigation, which covers nearly all bird species of the world, combined with the high resolution, as we use a direct measure (user derived through a web application) of bird aesthetic attractiveness to people. The latter represents a large improvement, as so far studies at such a large taxonomic scale could only use indirect proxies for human preference, such as number of likes for a specific species in Instagram^14^, or the occurrence of the species in social platforms^15^.

Human cognition and evolution as well as our socio-ecological history can contribute to understand our aesthetic preferences^14,16,17^. The ecological valence theory provides an explanatory framework based on both the evolutionary adaptive underpinnings of color preference as well as its emotional premise based on our past experience^18^. For example, blues and cyan are often associated with positive feelings related to a clear sky or clean water^16^, while red is connected to both positive and negative affective reactions, which may depend on the experience, from pleasant (e.g., sugar-rich red berries) to unpleasant (e.g., blood)^18^. More generally, red is associated with arousing signals in humans^19^, as well as a sign of high caloric content in food (such as red meat^20^). Moreover, humans are known to prefer foods of vivid colors, as they are fresher compared to dull colored (e.g., brown or grey) rotting foods^21^. Moreover, humans are also generally more attracted by multi-colored objects^22^, and by extravagant objects that depart from the usual shapes^23^. Therefore, we expect that blue and red colors, as well as more vivid colors (those departing from dull brown-grey; hereafter termed color elaboration), higher color-diversity and the presence of ornamental features (note that here, ornaments refers to the relative size of physical structures, such as crest, tail and beak, and not to colors) would drive our positive visual attraction for particular birds. Humans also find rare features generally more appealing, as the theory of negative frequency-dependent selection postulates^24^. Therefore, in addition to higher attraction to rarer color, we also predict that rarer features in birds, such as an extraordinary long beak, tail or crest, would make a bird more aesthetically attractive to humans. Finally, we expect that highly aesthetically attractive birds are also more threatened, based on recent evidence suggesting that colorful birds are more targeted for wildlife trade^7^.

## Results & discussion

**Fig. 1:**
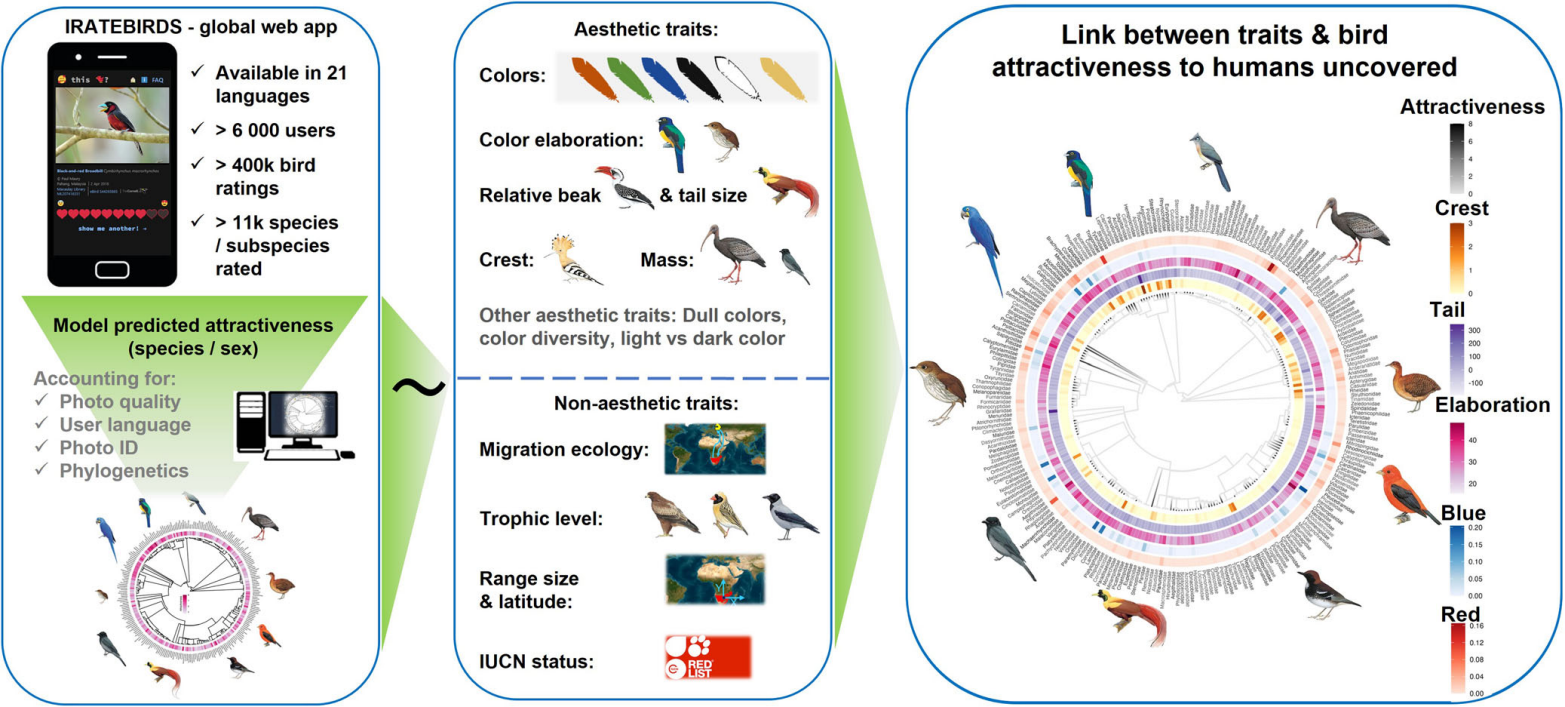
Workflow and most important variables used to assess variation in human visual attractiveness of birds. Scores of visual bird attractiveness (left) were obtained from a web application shared with users worldwide to rate the attractiveness of each bird species. These ratings were used to estimate average species by sex attractiveness scores, while accounting for factors such as photo quality and user language. The predicted bird attractiveness scores were then related to aesthetic (e.g., colour, colour elaboration, ornaments such as beak, tail and crest length, and body mass) and non-aesthetic traits (e.g., migration ecology, trophic level, range size and distribution, and threat status; central panel). We then modelled bird visual attractiveness to quantify which of the above traits make a bird more attractive to humans. The bird illustrations are reproduced with permission of Lynx Editions/Cornell Lab of Ornithology.

**Fig. 2:**
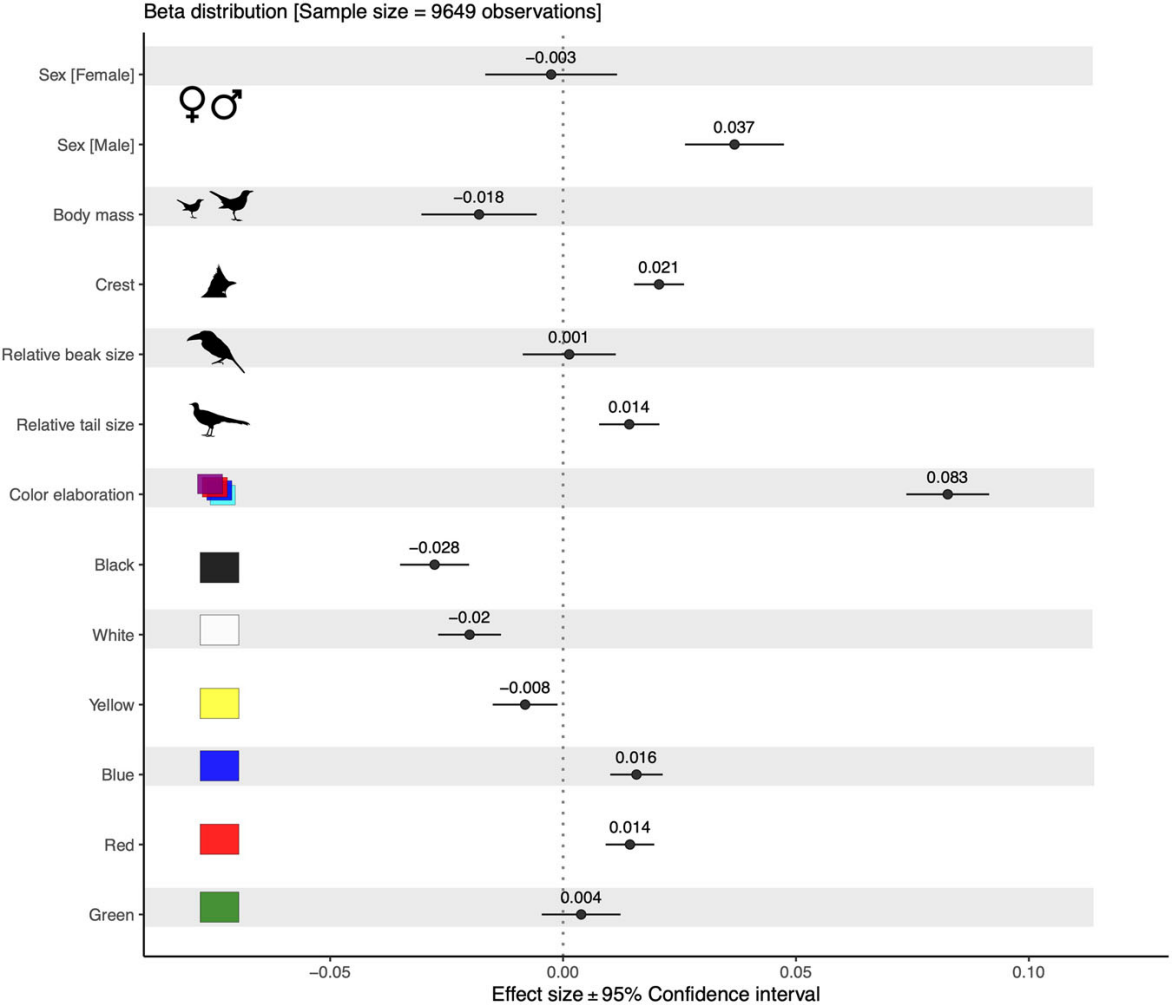
Global drivers of bird visual attractiveness. Estimated parameters (effect size mean ± 95% CI, *N* = 9649) from a beta generalized linear mixed-effect model (Eq. 1; see Supplementary Table 1 for details). Sex was the only categorical variable, with “undefined sex” set as the reference class. Body mass (in grams) was log transformed. Crest refers to a score but is considered a continuous variable (from absent to long crest). Beak and tail size represent the length, in mm, relative to body mass. Colour elaboration indicates the extent to which the overall colour of the bird departs from the global average across all birds (which is brown-grey). The six colour variables represent the proportion of the body covered by each colour. All continuous variables were scaled to a mean of zero and a standard deviation of one to allow direct comparison of effect sizes.

Here we combine a global citizen-science database on human-derived visual aesthetic attractiveness scores of nearly all bird species of the world^13^ with data on species-specific aesthetic traits, including coloration, size, size of ornaments (e.g., crest, beak, or tail), as well as non-visual traits such as range size, IUCN conservation status, migration ecology, and trophic level (Fig. 1). We model visual aesthetic attractiveness in relation to these traits using generalized linear mixed effects models that control for the effect of sex and taxonomy. The latter is important because visual aesthetic attractiveness to humans may be higher for the more ornamented sex in sexually dichromatic species^25^. Among 8852 species distributed across most of the world’s geographical realms, we identified several features that are associated with high visual aesthetic attractiveness to humans (Fig. 2 and Supplementary Table 1). (1) Attractiveness was associated with more red and blue plumage colours (especially light blue; Supplementary Fig. 1), and less black, white, and dull colours (brown and grey; Supplementary Fig. 2b). (2) Species with elaborated plumage colours (Fig. 2; defined as those colour combinations departing from dull brown-grey, see Methods) or multicoloured species (Supplementary Fig. 2a) were also scored as more attractive. (3) Attractiveness was further associated with the presence of ornaments, specifically with longer crests and longer tails. (4) Smaller species were more attractive than larger species. Finally, among the non-aesthetic traits, (5) species with larger range size were more attractive.

Our results are qualitatively robust to different quality checks, namely (i) re-running the analysis using three subsamples of data based solely on photos of males (*N* = 2531 species), females (*N* = 1012 species), or of undefined sex (*N* = 6070 species; largely sexually monomorphic species; Supplementary Fig. 3); and ii) re-running the analysis focusing only on the effect of the dark- or light-version of red, blue, and green colors (Supplementary Fig. 1). We note, however, that patterns of traits relationship to attractiveness are more evident for males than for females, possibly due to lower sample size and less variability in traits in females.

In line with our expectations, we found strong aesthetic preference for blue birds and birds with more elaborated colors (i.e., colorations departing from brown/grey; Fig. 2), and least preference for brown and grey (Supplementary Fig. 2b). These findings confirm the potential generality of the ecological valence theory as it may apply across the world’s birds. Similar aesthetic preferences for blue, obtained via questionnaires, have been previously reported for a selection of largely non-passerine bird families^11^, and preference for blue and green has been shown in a study focusing on pittas (Pittidae; see ref. ^22^). Moreover, Instagram photographs with birds having more blue are more liked than those with yellow, red or green^14^, a pattern that is at least partly confirmed in our study. We also show a strong preference for red birds, which contrasts with previous findings from studies on human preference for birds (e.g., see ref. ^22^). Beside colour, we also find a strong aesthetic preference for small birds. This result confirms a pattern that was previously reported in a study on Australian birds^12^.

A separate model which included colour diversity (see definition in methods) instead of the individual colour variables (necessary due to collinearity with blue, red, and green; Supplementary Fig. 4) showed that birds with multi-colour plumages were most attractive (Supplementary Fig. 2a). This aesthetic preference for colour diversity in birds aligns with recent findings from other studies showing that higher colour diversity is positively linked to the aesthetic value (e.g. in reef fishes^26^, Australian birds^8^, and imaginary animals^27^).

Humans are typically attracted by unusual, extravagant things that stand out in the crowd^23^. In the case of birds, ornamented species (e.g., with a long tail or long crest), or species which are more colourful and brighter coloured^8,12^ are more attractive to humans. Furthermore, species with brighter (lighter blue, see Supplementary Fig. 1) and more elaborated colours (unusual colours departing from brown-grey) are the most attractive to humans (Fig. 2). Overall, such findings of higher attractiveness for birds with less common features, be it ornaments or colors, also align with the negative frequency-dependent selection theory in humans^24^. The latter postulates that a preference for rare phenotypes is a type of selection that contributes to maintain rare alleles from being lost. This theory may, at least partly, explain the aesthetic preference we found for the rarest colors, like red and blue, and ornaments, such as long tail and crest. This is further supported by our result on the significance of color elaboration (i.e., rare colors departing from the average brown/grey), which was the variable with the strongest positive association to attractiveness among all those considered (Fig. 2). Further studies are needed to experimentally quantify the contribution of the above theories in shaping our aesthetic attractiveness for birds beyond the correlational evidence shown here (e.g., see ref. ^28^).

We also show that humans find birds with less black more visually attractive (Fig. 1), a pattern that deserves further investigations for it to be fully understood. In relation to colors, an issue we have not captured and that might deserve further attention is how the combination of colors on the birds impacts aesthetic preferences. For instance, harmony or similarity of colors in a bird’s plumage might impact our perceptions of it^29^.

Our analyses show that species with larger range size and distributed at higher latitudes scored as more attractive to humans (Supplementary Fig. 5). Previous studies showed that commonness strongly correlated with salience (measured as internet interest^15,30^ or scientific interest^9^, suggesting that we tend to find familiarity appealing. That birds at higher latitudes are scored as more attractive may also be explained by familiarity, given that most of the attractiveness scorers are from temperate and higher latitudes. The importance of familiarity underscores a potential challenge for conservation: the species that we may find less appealing because they are range restricted and breeding in the tropics are typically those most in need of conservation efforts^31,32^.

It might be that in reality there are different ways in which commonness and rarity or previous knowledge interact with our perception of species attractiveness. We however acknowledge that while the measure of attractiveness used here accounts for the country of origin of the scorer, we did not directly quantify the connection between familiarity to a specific species and its scored attractiveness. This topic thus deserves further investigations that span beyond the scope of this study.

Moreover, as the ecological valence theory proposes, our perception of objects (including animal species) is forged by the compilation of previous experiences and knowledge on the shape and colours, and the same most likely applies to birds^33,34^. Here, however, we did not aim to separate those previous experiences, but to study if, regardless of what those experiences are to each person, there are common drivers for the aesthetic attraction of birds to humans. Also, even if demographic variables and the bias towards respondents from certain countries should not be of importance in the metric we used, it is possible that the underlying attractiveness data used here did not fully capture all respondents from different cultures^13^.

Contrary to our expectation, we did not find any evidence that threatened birds would be more attractive than non-threatened ones (Supplementary Table 5). This result apparently contrasts with recent findings that colourful birds are more targeted, and thus potentially threatened, by wildlife trade^7^. Such discrepancy may be well explained by the fact that our measure of aesthetic attractiveness captures more factors at play than just the bird colors which are the focus of^7^. Also, we here considered all threats to birds, while in^7^ they exclusively focused on wildlife trade, making the results of the two studies not directly comparable. Another difference is that our analyses refer to the entire bird radiation while^7^ only considered passerine birds (Order Passeriformes). More in-depth analyses are needed to conclusively ascertain a possible relationship between bird aesthetics and threat status, possibly expanding from visual to also auditory cues, as it is well known that bird songs play a role in trade^35^.

Aesthetics permeate our life and are deeply rooted in the way we interact with the modern world^36^ and the environment^4,5^. For example, the impact of aesthetic preferences is evident in our choices for what we buy (the so-called “aesthetic fidelity effect”^37^), and in other spheres, such as science, where they affect what species we preferentially study, monitor, and protect^9,38^. Furthermore, aesthetic factors and charisma play a central role in our selection of conservation priorities, both for identifying species in need for conservation, or invasive species in need of eradication^39,40^. Likewise, among the public, people are generally more willing to pay for the conservation of aesthetically attractive species^41,42^. This underscores the wide, yet largely untapped cultural ecosystem service that aesthetics represent in conservation marketing, a relatively new and emerging field of science^43,44^.

While we unveil patterns that may help showcasing the potential of birds’ visual aesthetic value for their conservation, these same patterns may also represent a source of threat, as demand for traded birds is tightly linked to the birds’ aesthetical features, at least in terms of colour^7^. Ultimately, our findings can be used to emphasize the aesthetic value of birds to promote their conservation, and to provide early warnings of current and emerging threats from legal and illegal trade. Our results overall provide a general understanding on the role the different traits of birds play in shaping their aesthetic attractiveness. This knowledge will be key to appreciate and leverage the birds’ cultural ecosystem service to humans. Finally, such knowledge can also help to reduce the current research and conservation biases by highlighting least attractive species in need of higher attention both from the conservation and the scientific community, as well as the public.

## Methods

### Estimation of the attractiveness score for each species

We used a dataset of bird visual aesthetic attractiveness to humans covering almost all extant bird species^13^. We refer to^13^ for a detailed description of the database and associated methods. In brief, we generated this dataset through an internet-based application, the “iratebirds.app”, made available in 21 languages from different continents. We designed this application to allow users to rate the visual aesthetic attractiveness of a random selection of photographs of bird species. The user was asked to evaluate the looks of the bird with the text “please rate the appearance of this bird” on a scale from 1 to 10 of increasing attractiveness. We took all bird photos from the Cornell Lab of Ornithology’s Macaulay Library database. We used multiple photos per species or subspecies to minimize the impact of the individual photo (e.g., varying photo quality) on the final attractiveness score for each taxonomic unit. The Macaulay Library’s photo quality is rated by users [1 (low) – 5 (high)] based on the bird being clearly visible and sharply shown in the photo. To minimize the effect of varying photo quality, we prioritized the use of the best available photographs by randomly choosing at least five of the top user-rated photographs per species. In case less than five photos of a species were available, we used all photos, and in some cases, more have been used (mean = 5.2 photos per species, range = 1–15).

Overall, more than 6000 respondents from 78 countries, mainly from the Western cultural sphere (e.g., Finland, Russia, Italy, U.S.A, Japan, France, Spain based on information from the 2738 participants who gave their demographic information), scored photos in the application. Spreading the questionnaire electronically via social media, email lists and other media, e.g. newspapers, did not allow for control of the sampling of the participants. However, we had a good distribution of nearly all demographic variables besides home country (e.g., both birders and non-birders, young and old people, people with strong nature related experience and knowledge of birds, and those without e.g. lacking much prior knowledge of birds^13^). This yielded a total of > 400,000 scores for > 11,000 bird taxonomic units (species and subspecies). We used these scores to predict visual aesthetic attractiveness of each species and subspecies. We fitted a regression model to the data, designed to estimate an aggregate consensus score for each species (and sex for dichromatic species). To take into account confounding factors that could have affected the attractiveness score, we included two covariates in the model: (1) variation in the quality of the photograph (score 1–5), and (2) user language (“culture”). To avoid pseudo-replication due to the same photograph or species being scored multiple times and to account for phylogeny^13^, we included photo identity, species, genus, order and family as random factors. For dichromatic species, we obtained sex-specific scores, based on information provided by the authors of the photographs about the sex of the bird. Thus, we also included sex in the model (three categories: male, female, unknown sex^13^). We compared the above-derived estimates for each species with those based on a subset of the data modeled as above but with the addition of other potential user-specific confounding factors: home country, age, birdwatching activity, environmental awareness and nature-related attitudes^13^. These variables (and the attractiveness scores) were available for those users who answered the application’s background survey on demographic data and nature- and bird-related attitudes and knowledge (*N* = 2785, 45% of all users^13^). The two sets of estimates were strongly correlated (*r* = 0.92), suggesting that the second set of potentially confounding variables had little influence on the visual aesthetic attractiveness estimates.

For each species, we obtained either the average visual aesthetic attractiveness estimate (e.g., for sexually monomorphic species), or a separate estimate for males and/or for females (for sexually dichromatic species). The final database consisted of a total of 11,187 visual aesthetic attractiveness estimates at the species and sex level. For 7060 species, we only obtained average attractiveness estimates, and for 2963 and 1164 species we obtained separate estimates for males and females, respectively.

### Predictor variables related to visual aesthetics

We assembled a dataset of phenotypic traits that are potentially related to the visual aesthetic attractiveness of birds to humans. These variables include coloration (e.g., amount of red, blue, and green), color elaboration (i.e. how much the overall plumage departs from a dull brown-grey coloration), size, and length of crest, tail or beak (these latter three variables are here referred as ornaments).

Color has been previously found to be a central determinant of bird aesthetic attractiveness to humans^11,12,22^. We computed color variables for each species and sex (when available) based on the digitised illustrations from the Handbook of the Birds of the World (available at www.birdsoftheworld.org and described in^45,46^). Red, Green, and Blue (RGB) pixel values in each image were converted into CIELAB visual space, which is perceptually uniform for humans, and has two chromatic (a, b) and one achromatic (L) axis of variation (Supplementary Fig. 6a). To reduce the dimensionality of the data we first defined 12 colour categories. To achieve this, we used a three-dimensional grid that split the chromatic axes (a, b) into 12 bins and the achromatic axis (L) into 4 bins. This yielded a total of 576 cells of which 216 were occupied by colours in the sample (Supplementary Fig. 6a, b). For each image we computed the proportion of pixels falling into each of these bins using the function getLabHist from the R package *colordistance* version 1.1.2. As shown previously^47^, most colours are concentrated towards the centre of the colour space and colours away from the centre become progressively rarer (Supplementary Fig. 6). To further reduce dimensionality, we combined adjacent cells to create a set of 12 categories, and thus for each image we obtained the proportion of the body covered by blue, purple, red, yellow, green, rufous, brown, grey, white, and black (Supplementary Fig. 6b). The most common colour categories were grey, black, white, and brown, while other colours were much rarer and generally comprised less than 1% of the body.

Because humans typically prefer lighter colours compared to dark ones^11,48^, we also separated between the light and dark form of each of the following colours: blue, brown, green, grey, purple, red, and rufous (see Supplementary Fig. 6b). Due to the way white, black and yellow were defined, it is not meaningful to separate the dark and light version.

Colour diversity, as well as extreme colours departing from the average, have been positively related to bird attractiveness^12^. Therefore, we calculated a measure of colour diversity and colour elaboration. Colour diversity was defined as the number of occupied colour loci^31,47^, computed as the total number of occupied cells in the grid described above (with an average of 48, and varying between 12 and 141). We computed colour elaboration as the average distance between all colours found in a species and the global average colour^43^ across all species (which is brown-grey). This trait varies between 12 (e.g., in *Stresemannia bougainvillei*) and 57 (e.g., in a male *Ploceus bojeri*), whereby species with lower values of colour elaboration have mostly “duller” cryptic colours. All colour variables were calculated for each sex separately, except for sexually monomorphic species and for those where illustrations for one of the sexes were missing, following previous studies^45^.

As a measure of size, we used data on species-specific body mass, obtained from the AVONET database on bird traits^32^. We obtained measures for other potential ornamental traits, such as crest, beak, and tail length. Based on illustrations^49^, we scored crest length for each species and sex (if illustrations available), using four discrete categories: 0 = no crest, 1 = crest follows shape of head, 2 = short crest, and 3 = long crest (see Supplementary Fig. 7). We obtained data on beak length (length from the tip of the beak to the base of the skull) and tail length (distance between the tip of longest rectrix and the point at which the two central rectrices protrude) for each species from^32^. In the analyses, we used length relative to size (body mass) by including the residuals of a regression model of beak or tail length against body mass.

### Predictor variables unrelated to visual aesthetics

We considered non-visual traits that may also underlie bird aesthetic attractiveness: range size (a proxy for species familiarity), International Union for Conservation of Nature (IUCN) threatened status (a proxy for rarity), trophic level, migration ecology, and latitude of the species range.

For each species, we defined range size as the size of the resident or breeding range, which we extracted from^32^. We extracted the threat status from the IUCN Red List of Threatened species^50^, but reclassified this variable into three categories to balance factor levels: Threatened (including ‘Vulnerable’, ‘Endangered’ and ‘Critically Endangered’ species), Non-threatened (including ‘Least Concern’ and ‘Near Threatened’ species), and Unknown status (including ‘Data Deficient’ and ‘Not Evaluated’ species). We obtained data about the species-specific trophic level from^32^ and reclassified it into three categories: carnivores (including all predators, scavengers, and invertivores), omnivores, and herbivores. Similarly, we obtained information about the migratory status of each species^32^ and reclassified the data into two categories: migratory species (either partial or full migrant) and non-migratory species (resident). To account for potential latitudinal patterns in the data^31^, we obtained the latitude of the centroid of the range for each species from^32^.

### Data analysis

We ran all analysis in R version 4.1.0^51^, using the *tidyverse* suite^52^ for data handling and visualizations. In regression analyses, we followed the general protocol from^53^. In interpreting results, we used an evidence-based language^54^, whereby we focused on effect sizes and direction of effects rather than significance (i.e., *p*-values). We however report exact *p*-values in Supplementary Tables 1–5 and in Fig. 2.

### Data exploration

Prior to model construction, we visually inspected variable distribution, presence of outliers, multicollinearity among predictors, and balance of factor levels^53^. As a result, we log-transformed body mass and range size to homogenize their distribution and minimize the effect of a few outliers. Furthermore, we scaled (to a mean of zero and a standard deviation of one) all continuous variables to obtain comparable effect sizes and facilitate convergence of regression models. Following multicollinearity testing with Pearson’s *r* correlations, we dropped the variable colour diversity (Supplementary Fig. 4), because it correlated (|*r* | > 0.6) with several other colour variables, and tested its effect in a separate model (Supplementary Fig. 2a). Most colour variables were also correlated (Supplementary Fig. 4), so we kept six uncorrelated colours in the model (black, white, yellow, blue, red, and green), capturing most of the chromatic variability across birds. Note also that the excluded colours, such as purple, brown, grey and rufous, represent colours closer to the global colour average across all species, meaning that these are well captured as a group by the lowest values of the colour elaboration variable. We tested the effect of these excluded dull colours in a separate model (Supplementary Fig. 2b). Pearson’s *r* correlations among the final set of predictors were all below ± 0.5 (Supplementary Fig. 8). In the main text, we present results using the sum of the light and dark version of each colour (Fig. 2), but we also analysed light and dark colours separately (Supplementary Fig. 1).

### Regression analyses

We built generalized linear mixed effects models using the R package *glmmTMB* version 1.1.1^55^, assessing the relationship between species traits (independent variables) and attractiveness (dependent variable). The sampling unit for these models is at the level of the species, or sex within the species (in the case of dichromatic species). Since the response variable is a continuous score between 0 and 9 (converted from the original score range of 1 to 10), we divided the variable by 10 and modelled it using a beta error distribution. The structure of the model, in R notation, was:

1 $$\begin{array}{l}{Attractiveness} \sim {sex}+{body}\,{mass}+{crest}+{relative}\,{beak}\,{length}\\ \qquad\qquad\qquad\quad+\,{relative}\,{tail}\,{length}+{colour}\,{elaboration}+{black}\\ \qquad\qquad\qquad\quad+\,{white}+{yellow}+{blue}+{red}+{green}+(1{\rm{|}}{Order})\\ \qquad\qquad\qquad\quad+\,(1{\rm{|}}{Family})+(1{\rm{|}}{Genus})+(1{\rm{|}}{Species}\,{ID})\end{array}$$

We included Order (factor with 41 levels), Family (247 levels) and Genus (2248) as random intercept factors to account for taxonomic non-independence of samples. Furthermore, we included Species ID (8852 levels) as a random factor to account for pseudo-replication stemming from repeated measures of the same species when the attractiveness of two sexes was scored.

We carried out model validation by inspecting model residuals with the *check_model* function in the package *performance* version 0.9.0.6^56^.

We repeated the above main model by adding other non-aesthetic variables, such as trophic level, IUCN status, range size, latitude and longitude, and migratory status:

2 $$\begin{array}{l}{Attractiveness} \sim {sex}+{trophic}\,{level}+{IUCN}+{range}\,{size}\\ \qquad\qquad\qquad\quad+\,{\rm{|}}{latitude}{\rm{|}}+{migration}+{body}\,{mass}+{crest}\\ \qquad\qquad\qquad\quad+\,{relative}\,{beak}\,{length}+{relative}\,{tail}\,{length}+{colour}\,{elaboration}\\ \qquad\qquad\qquad\quad+\,{black}+{white}+{yellow}+{blue}+{red}+{green}+(1{\rm{|}}{Order})\\ \qquad\qquad\qquad\quad+\,(1{\rm{|}}{Family})+(1{\rm{|}}{Genus})+(1{\rm{|}}{Species}\,{ID})\end{array}$$

We ran two additional sets of models to check the consistency of our results. First, we ran three models using three subsamples of data based solely on observations of males (*N* = 2531), females (*N* = 1012), or undefined sex (*N* = 6070; including largely sexually monomorphic species) (Supplementary Fig. 3). These three models were the same as the one described in Eq. 1, but excluding the term sex and the random factor Species ID. Next, we ran two models focusing only on the effect of the dark or light version of the colours red, blue and green (for other colours, such as yellow, white and black it is not meaningful to separate the light and dark version, as explained above). The structure of the model was the same as in Eq. 1, except that we used either the dark or light colour variable (rather than their sum; Supplementary Fig. 1).

The number of loci (a measure of colour diversity) was highly correlated (*r* > 0.6) with other colour variables, such as red, green, purple and rufous (Supplementary Fig. 4). Similarly, the dull colours, such as grey, brown and rufous, in addition to purple, correlated strongly ((|*r* | > 0.5) with other colours (Supplementary Fig. 4). Because of this collinearity, we tested the number of loci, and separately the dull colours and purple, in a basic model as in Eq. 1, but with the colour variables replaced by either the number of loci or by the dull colours and purple (results presented in Supplementary Fig. 2).

We present the results as model-derived effect sizes (Fig. 2 and Supplementary Table 1), and we also report the significant (as in Fig. 2) aesthetic variables as well as raw attractiveness scores at the family level (Supplementary Fig. 9), along with the model-derived residual variation (from the main model presented in Fig. 2) at the level of bird orders (Supplementary Figs. 10, 11).

### Supplementary information

Supplementary information

## Data Availability

All data used in this study are openly available in Figshare repository at: https://figshare.com/s/5602ec236f40db0eb162.

## Code availability

All codes/scripts used to conduct the analyses are detailed and openly available in Figshare repository at: https://figshare.com/s/5602ec236f40db0eb162.
