# Supplementary material for: What drives our aesthetic attraction to birds?
Source: NPJ Biodivers. 2023 Sep 27;2:20. doi: 10.1038/s44185-023-00026-2 (PMC11332239; doi:10.1038/s44185-023-00026-2)
Supplement: Supplementary file 1 — Supplementary information [file 44185_2023_26_MOESM1_ESM.pdf]

What drives our aesthetic attraction to birds?

Authors: Santangeli Andrea, Anna Haukka, William Morris, Sarella Arkkila, Kaspar Delhey, Bart Kempenaers, Mihai Valcu, James Dale, Aleksi Lehikoinen, and Stefano Mammola

Supplementary Tables:

Supplementary Table 1: Main model results as presented in Fig. 2

Variable	Estimate	SE	z	p
Sex [Female]	-0.003	0.007	-0.354	0.723
Sex [Male]	0.037	0.005	6.788	0.000
Body mass	-0.018	0.006	-2.855	0.004
Crest	0.021	0.003	7.519	0.000
Relative beak size	0.001	0.005	0.260	0.795
Relative tail size	0.014	0.003	4.302	0.000
Color elaboration	0.083	0.005	18.203	0.000
Black	-0.028	0.004	-7.299	0.000
White	-0.020	0.003	-5.834	0.000
Yellow	-0.008	0.004	-2.296	0.022
Blue	0.016	0.003	5.483	0.000
Red	0.014	0.003	5.384	0.000
Green	0.004	0.004	0.899	0.369

Supplementary Table 2. Estimated regression parameters and p-values from the models same as that presented in Figure 2 of the main text but this time testing the effect of dark (left) and light (right) blue, red and green colors on bird visual attractiveness (the response variable).

	Dark RGB model					Light RGB model			
**Variable**	**Estimate**	**SE**	**z**	**p**	**Variable**	**Estimate**	**SE**	**z**	**p**
Sex [Female]	-0.002	0.007	-0.317	0.752	Sex [Female]	-0.003	0.007	-0.417	0.677
Sex [Male]	0.038	0.005	6.920	0.000	Sex [Male]	0.038	0.005	6.934	0.000
Body mass	-0.018	0.006	-2.794	0.005	Body mass	-0.019	0.006	-2.972	0.003
Crest	0.021	0.003	7.480	0.000	Crest	0.021	0.003	7.594	0.000
Relative beak size	0.002	0.005	0.312	0.755	Relative beak size	0.002	0.005	0.350	0.727
Relative tail size	0.014	0.003	4.194	0.000	Relative tail size	0.013	0.003	4.032	0.000
Color elaboration	0.089	0.004	22.538	0.000	Color elaboration	0.093	0.004	22.986	0.000
Black	-0.032	0.003	-9.227	0.000	Black	-0.033	0.004	-9.298	0.000
White	-0.023	0.003	-7.403	0.000	White	-0.026	0.003	-8.054	0.000
Yellow	-0.012	0.003	-3.680	0.000	Yellow	-0.015	0.003	-4.469	0.000
**Dark blue**	0.008	0.003	2.908	0.004	**Light blue**	0.014	0.002	5.472	0.000
**Dark red**	0.013	0.003	5.040	0.000	**Light red**	0.006	0.002	2.410	0.016
**Dark green**	0.004	0.003	1.408	0.159	**Light green**	-0.004	0.004	-1.091	0.275

Supplementary Table 3. Estimated regression parameters and p-values from the models same as that presented in Figure 2 of the main text but this time replacing in turn the color variables with n.loci (top), which represents the number of different colors of a specific bird (i.e. a measure of color diversity), or with the dull colors (bottom: brown, grey, purple, rufous) in their effect on bird visual attractiveness (the response variable).

N.loci Model:

Variable	Estimate	SE	z	p
Sex [Female]	-0.015	0.007	-2.058	0.040
Sex [Male]	0.048	0.005	8.769	0.000
Body mass	-0.019	0.006	-2.911	0.004
Crest	0.018	0.003	6.393	0.000
Relative beak size	0.005	0.005	0.934	0.350
Relative tail size	0.017	0.003	4.910	0.000
**N.loci**	0.074	0.003	23.003	0.000

Dull colors model:

Variable	Estimate	SE	z	p
Sex [Female]	-0.010	0.007	-1.305	0.192
Sex [Male]	0.045	0.006	8.211	0.000
Body mass	-0.019	0.007	-2.841	0.005
Crest	0.020	0.003	6.987	0.000
Relative beak size	0.004	0.005	0.666	0.506
Relative tail size	0.014	0.003	4.227	0.000
**Brown**	-0.036	0.003	-11.892	0.000
**Grey**	-0.055	0.003	-17.936	0.000
**Purple**	0.010	0.002	4.516	0.000
**Rufous**	0.008	0.002	3.345	0.001

Supplementary Table 4. Estimated regression parameters and p-values from the models same as that presented in Figure 2 of the main text but this time using in turn a subset of the data based only on male (left), female (center), or undefined sex (right; an average across sexes, relating to the monomorphic species). Response variable is the bird visual attractiveness.

	Male				Female				Average			
**Variable**	**Estimate**	**SE**	**z**	**p**	**Estimate**	**SE**	**z**	**p**	**Estimate**	**SE**	**z**	**p**
Body mass	-0.012	0.011	-1.09	0.277	-0.010	0.016	-0.65	0.517	-0.027	0.008	-3.56	<0.001
Crest	0.018	0.005	4.00	<0.001	0.024	0.009	2.75	0.006	0.021	0.004	5.85	<0.001
Relative beak size	0.004	0.011	0.37	0.710	0.019	0.011	1.74	0.081	-0.001	0.006	-0.21	0.832
Relative tail size	0.011	0.005	2.18	0.030	-0.007	0.007	-0.99	0.321	0.025	0.005	5.23	<0.001
Color elaboration	0.062	0.009	6.89	<0.001	0.079	0.014	5.59	<0.001	0.091	0.006	16.11	<0.001
Black	-0.020	0.007	-2.88	0.004	-0.025	0.013	-1.99	0.047	-0.034	0.005	-6.96	<0.001
White	-0.006	0.007	-0.96	0.337	-0.015	0.011	-1.39	0.164	-0.025	0.004	-5.84	<0.001
Yellow	-0.005	0.007	-0.73	0.463	-0.026	0.011	-2.43	0.015	-0.006	0.004	-1.42	0.154
Blue	0.017	0.005	3.65	<0.001	0.018	0.014	1.26	0.206	0.015	0.004	3.81	<0.001
Red	0.017	0.004	4.04	<0.001	0.014	0.012	1.16	0.244	0.015	0.004	3.92	<0.001
Green	0.022	0.007	3.08	0.002	0.007	0.011	0.69	0.492	0.003	0.006	0.52	0.604

Supplementary Table 5. Estimated regression parameters and p-values from the models same as that presented in Figure 2 of the main text but this time also including non-aesthetic trait variables (bold fonted), such as migration ecology (reference: "non-migratory"), trophic level (reference: "carnivore", which also includes insectivores), range size and latitude of the species distribution centroid, and IUCN conservation status (reference: "unknown").

Variable	Estimate	SE	z	p
Sex [Female]	-0,012	0,007	-1,69	0,091
Sex [Male]	0,026	0,005	4,75	<0,001
Body mass	-0,023	0,006	-3,62	<0,001
Crest	0,020	0,003	7,61	<0,001
Relative beak size	0,005	0,005	0,93	0,353
Relative tail size	0,013	0,003	3,95	<0,001
Color elaboration	0,087	0,004	19,49	<0,001
Black	-0,027	0,004	-7,31	<0,001
White	-0,028	0,003	-8,06	<0,001
Yellow	-0,011	0,003	-3,22	0,001
Blue	0,014	0,003	4,98	<0,001
Red	0,013	0,003	4,89	<0,001
Green	0,002	0,004	0,57	0,569
**Migratory [Yes]**	-0,009	0,007	-1,27	0,205
**Trophic level [Herbivore]**	-0,014	0,008	-1,65	0,098
**Trophic level [Omnivore]**	0,011	0,008	1,30	0,193
**Range size**	0,031	0,003	12,00	<0,001
**Latitude (range centroid)**	0,023	0,003	8,23	<0,001
**IUCN [Threatened]**	0,033	0,015	2,27	0,023
**IUCN [Not threatened]**	0,037	0,014	2,71	0,007

Supplementary Figure:

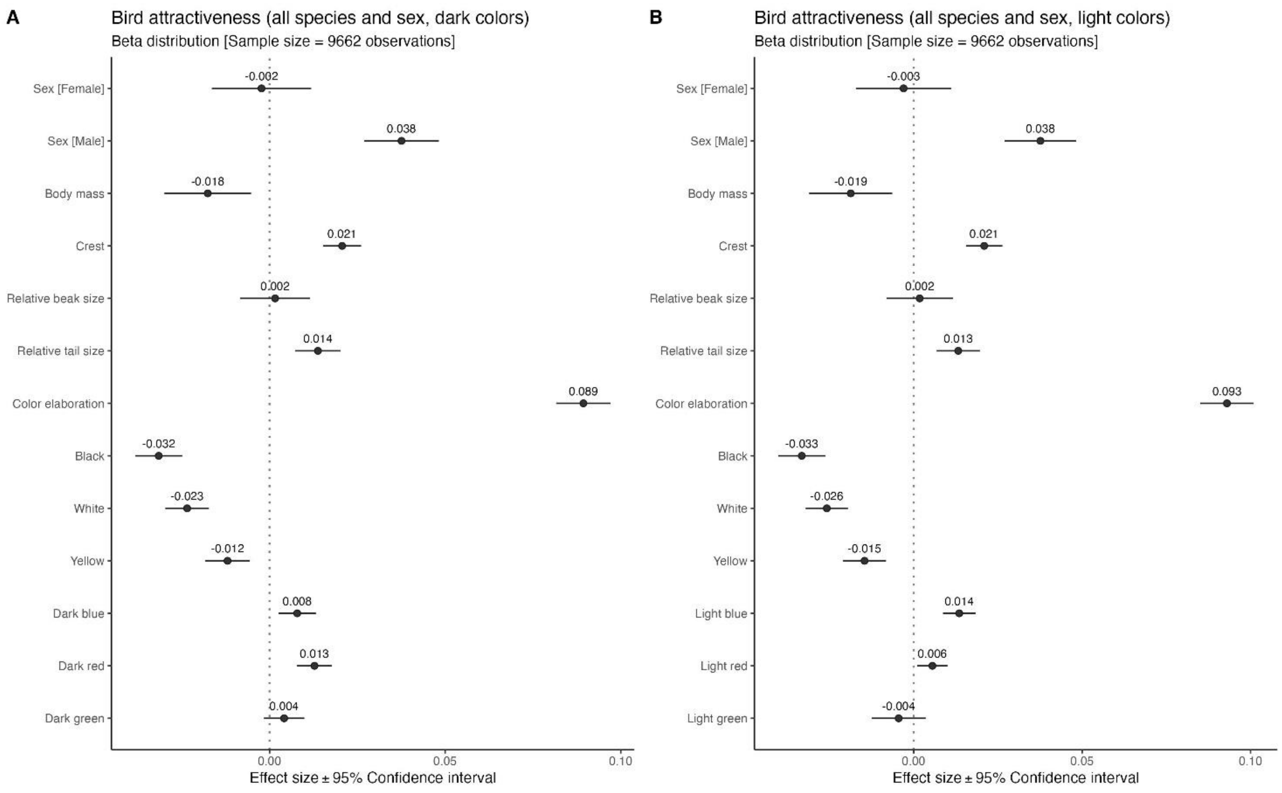

Figure 1. Drivers of bird attractiveness based on subset models considering the dark (A) and light (B) colors blue, red, and green. Estimated regression parameters and p-values are in Supplementary Table 2. Factors baselines: "average" for sex.

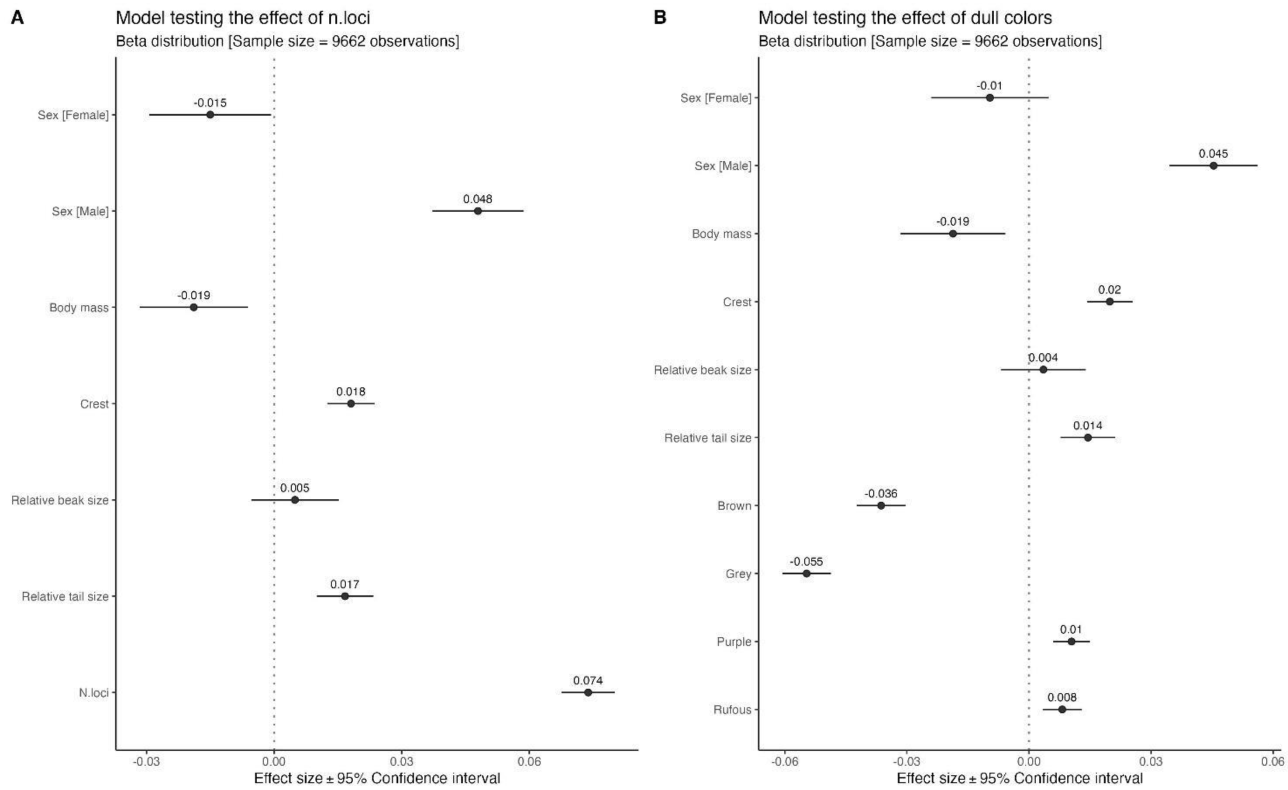

Figure 2. Drivers of bird attractiveness based on subset models considering the n. loci (A) as a measure of plumage color diversity, and dull colors (B), such as brown, grey, purple and rufous. Estimated regression parameters and p-values are in Supplementary Table 3. Factors baselines: "average" for sex.

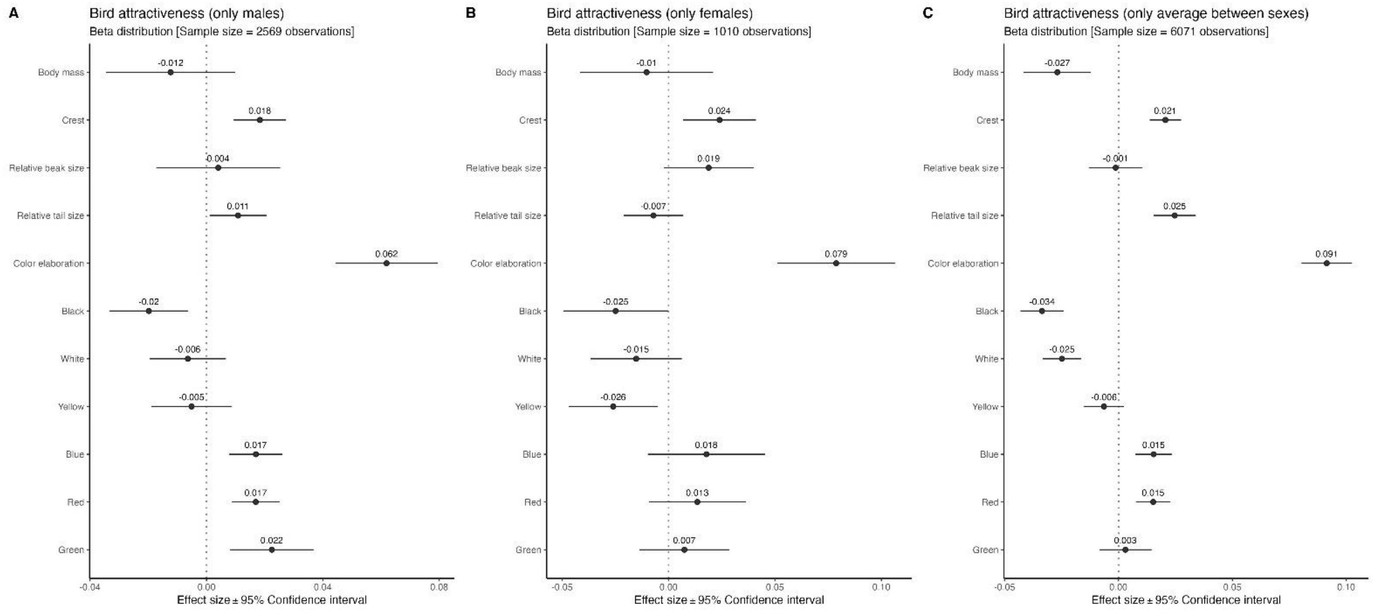

Figure 3. Drivers of bird attractiveness based on subset models considering observations of males only (A), females only (B), or the average between sexes (C). Estimated regression parameters and p-values are in Supplementary Table 4. Factors baselines: "average" for sex.

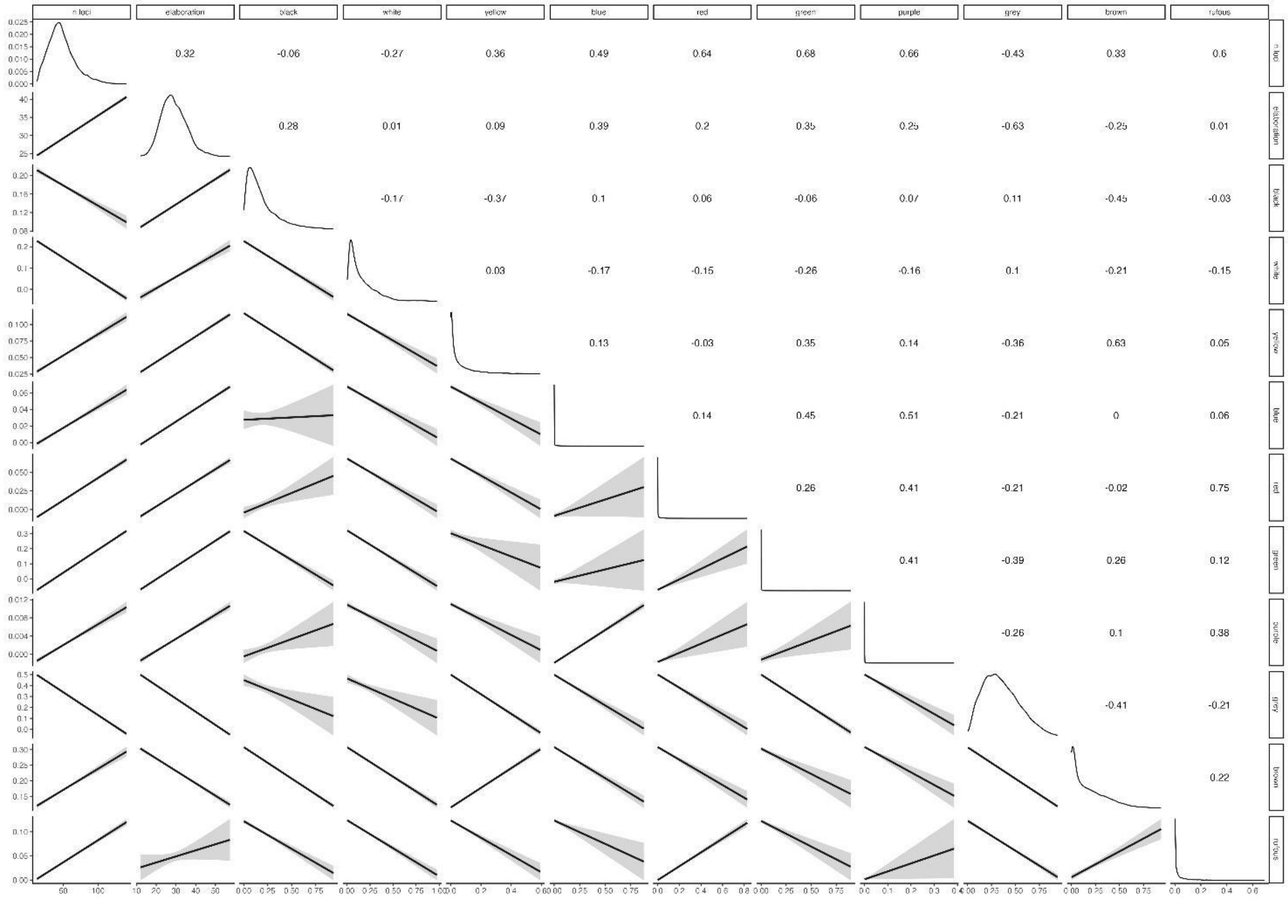

Figure 4. Collinearity value between all the color related variables considered for study. Specifically, the variables include overall color diversity (n.loci), color elaboration (elaboration), and ten variables each representing one color. See methods for details on each variable.

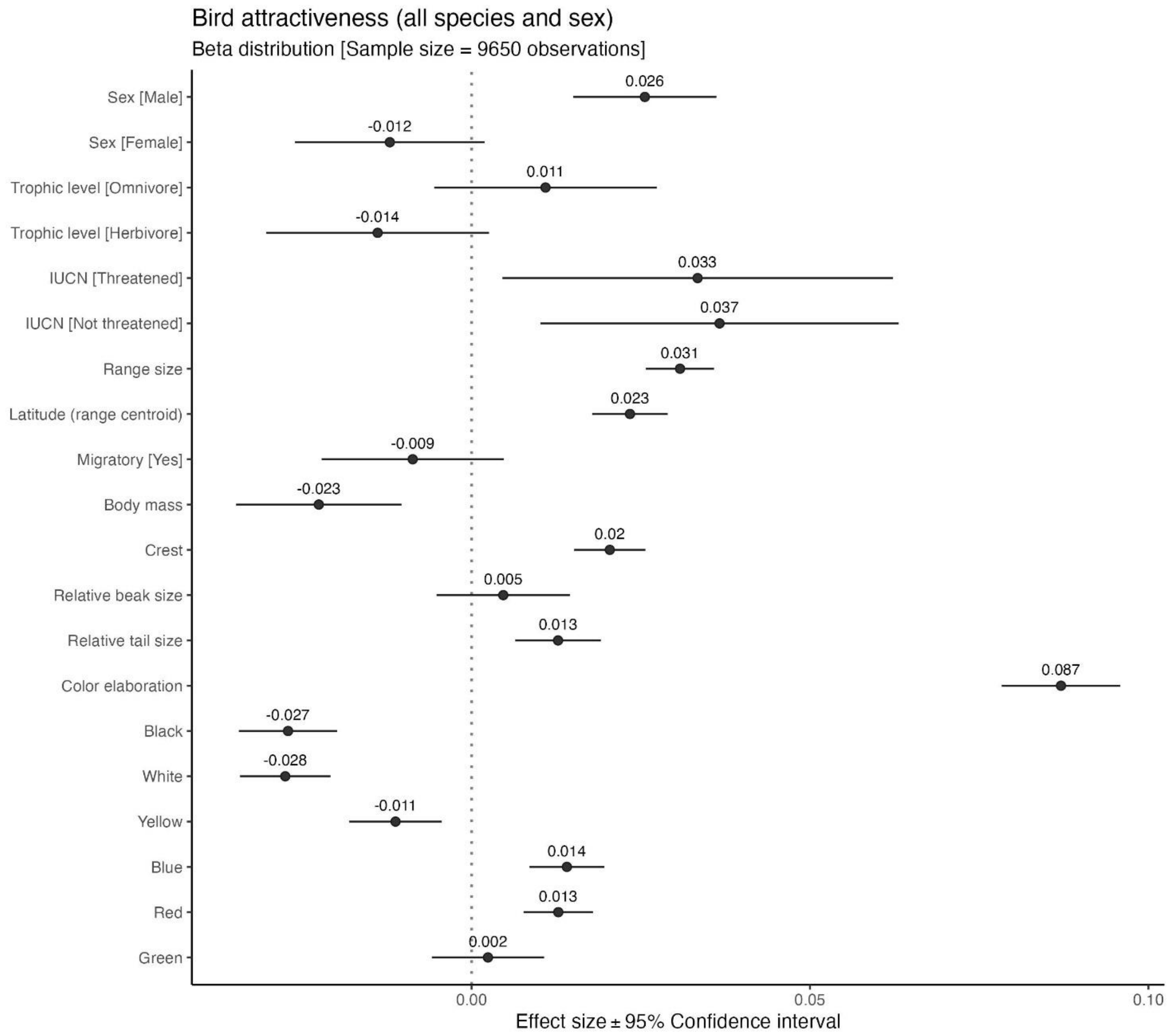

Figure 5. Results of a full model including both aesthetic and non-aesthetic traits (such as trophic level, IUCN status, range size, latitude and migration ecology). Estimated regression parameters and p-values are in Supplementary Table 5. Factors baselines: "Average" for sex, "Carnivore" for Trophic level, “Unknown” for IUCN, “Non-migratory” for migration. Latitude is expressed in absolute values, from low to high latitudes.

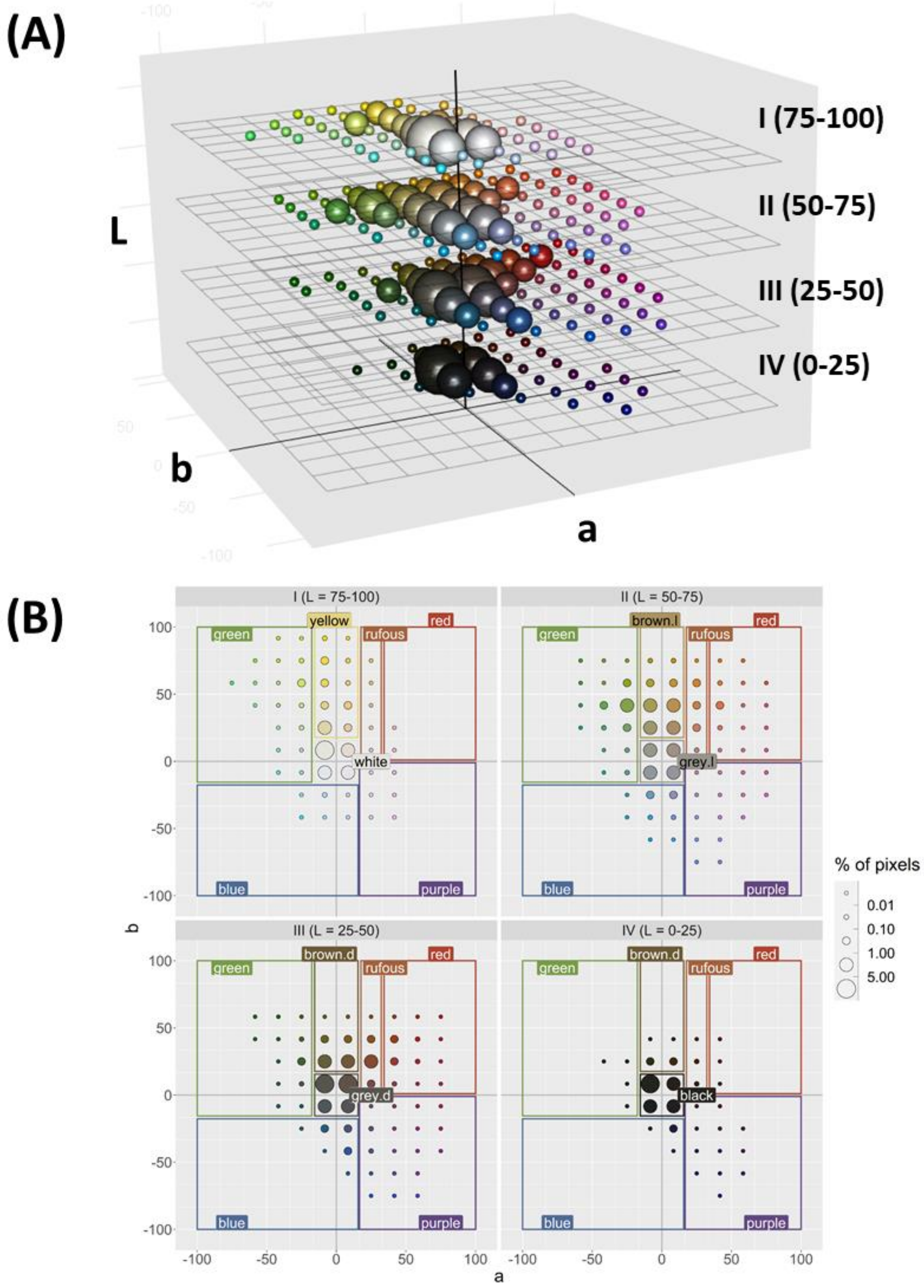

Figure 6. Assigning colours to broad categories. (A) Shows 216 binned colour categories in CIELAB colour space. Sphere size indicates the proportion of all colours in each bin. Bins are formed by a 3D mesh with 12 levels per chromatic dimension (a and b) and 4 levels for the achromatic dimension (L). To indicate the limits between colour categories we show each level along the L dimension (I-IV from light to dark in (A)) as a separate panel in (B). Limits around the different colour bins (circles, size is proportional to the % of all pixels within that bin) group them into separate colour categories as indicated by the labels. We used cells in achromatic levels I and II (light) and II and IV (dark) to separate light and dark versions of each colour category (except yellow, black and white).

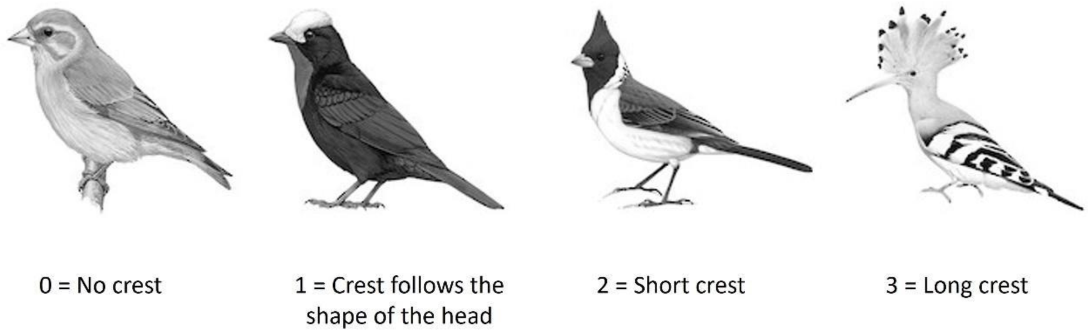

Figure 7. Example species for each of the four classes used to quantify the crest ornamentation, from no crest, to a small crest that follows the shape of the head, a small crest that departs from the shape of the head, and finally a long crest.

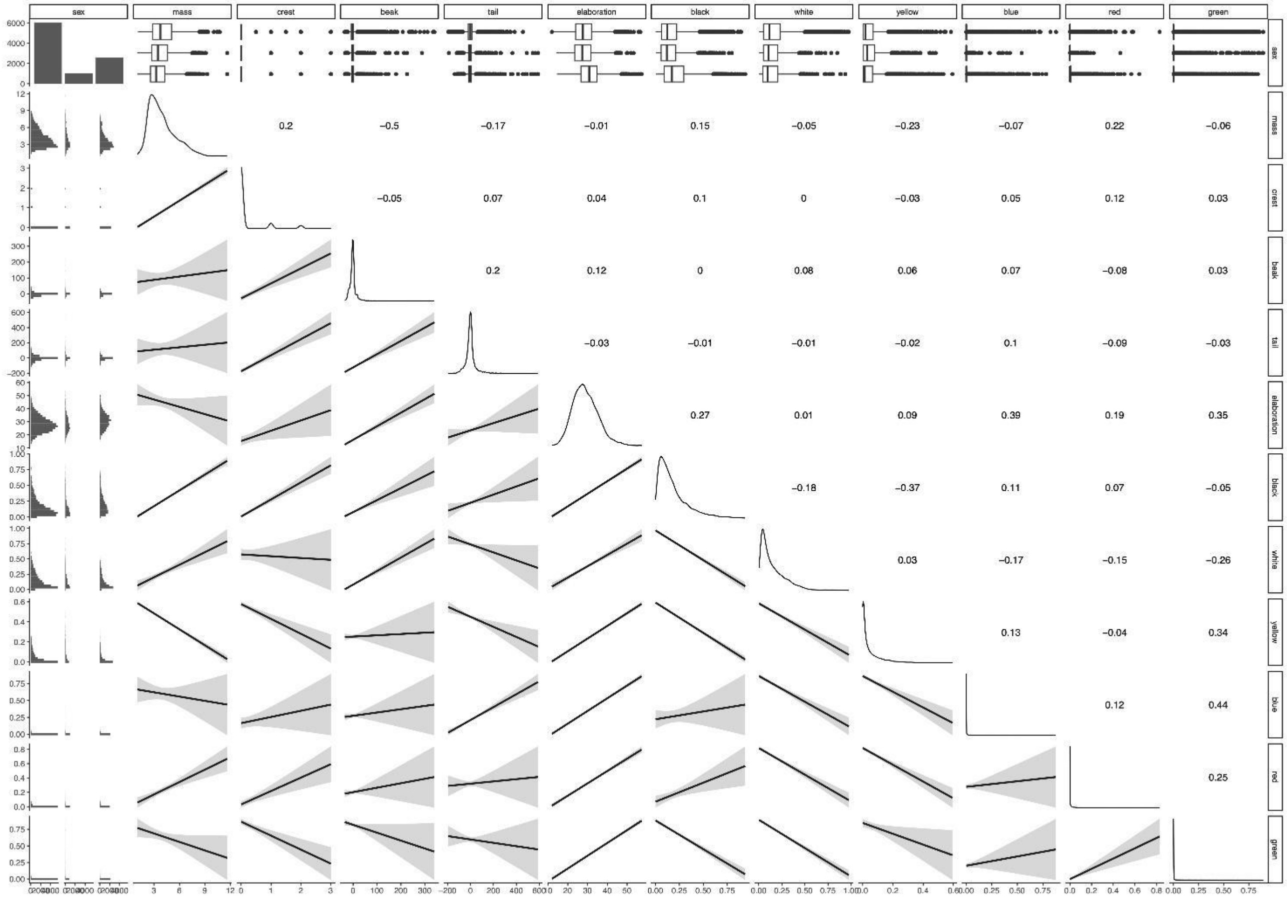

Figure 8. Multicollinearity among all predictors used in the main model as presented in Fig. 2 of the main text. On the diagonal: density plot showing variable distribution. Below the diagonal: regression lines between pairwise comparisons. Above the diagonal: Correlations are expressed as Pearson's r. Variables include n. loci (the number of discrete colors in a bird), elaboration (the amount of extreme coloration, i.e. departing from dull brown-rufous colors) followed by the 10 discrete colors considered initially for the study.

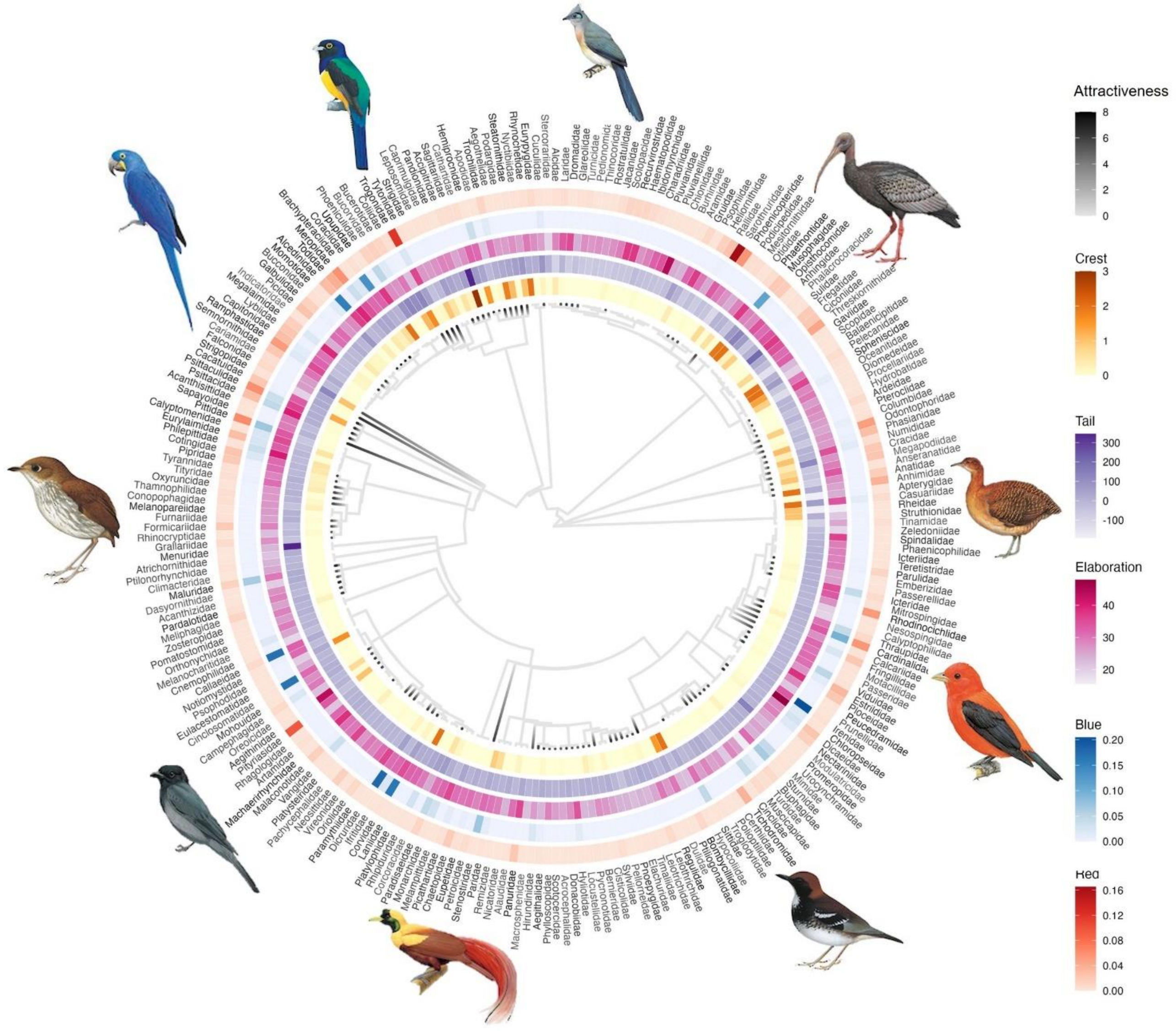

Figure 9. Visual attractiveness and other color traits across the worlds' birds. We present a summary of the birds visual attractiveness to humans (grey scale, inner circle bars) averaged across each of the bird families. We also present a summary of the five aesthetic trait variables with positive effect on bird visual attractiveness to humans. These include the crest length (from 0 = no crest to 3 = a very long crest), tail length (relative to the species body mass), color elaboration (i.e. how far the overall color of the bird departs from the dull brown-grey global average), as well as two plain colors, blue and red. The latter two represent the proportion of the body covered by each color. Illustrations represent 10 examples of bird species with contrasting attractiveness scores. Illustrations are reproduced with permission of Lynx Editions/Cornell Lab of Ornithology.

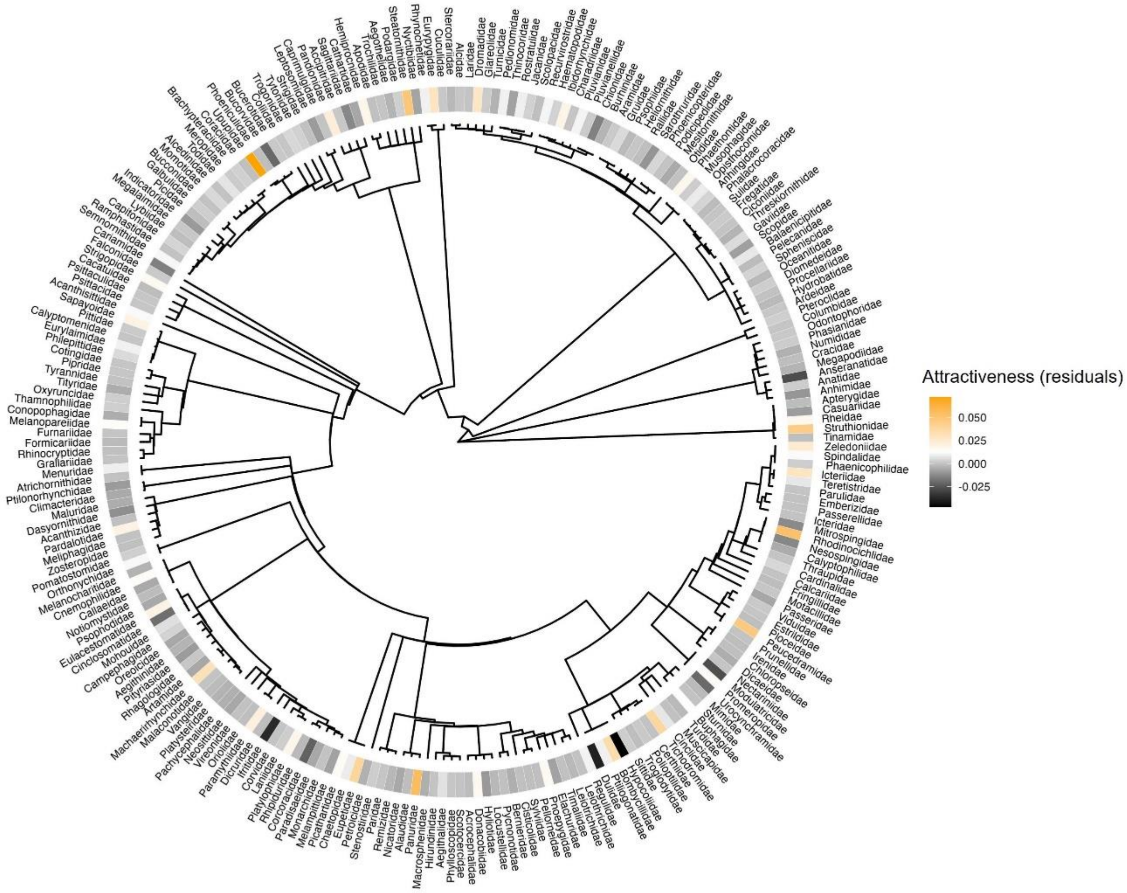

Figure 10. Residual attractiveness across the worlds' birds families after accounting for the effect of aesthetic traits on attractiveness. The values are derived from the residuals of the model whose results are presented in Fig. 2 of the main text. Positive values (towards dark yellow) depict bird families whose attractiveness is higher than expected given their aesthetic traits, and vice versa for negative (towards black) values.

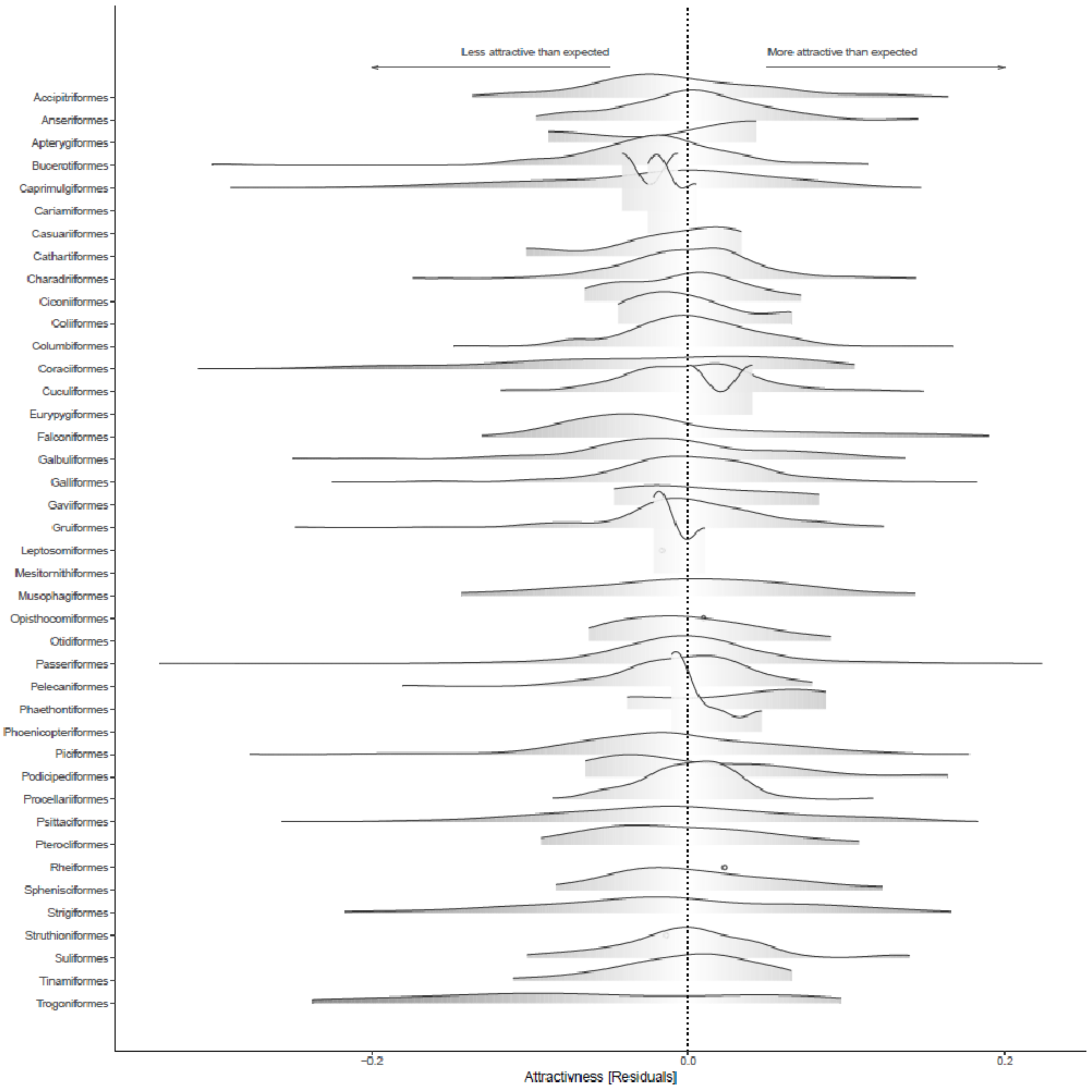

Figure 11. Residual attractiveness distribution across the worlds' birds orders after accounting for the effect of aesthetic traits on attractiveness. The density plots show the residual distribution as obtained from residual values from the model whose results are presented in Fig. 2 of the main text. Positive values (towards dark yellow) depict bird families whose attractiveness is higher than expected given their aesthetic traits, and vice versa for negative (towards black) values.
